# Deletion of Semaphorin 3F in Interneurons Is Associated with Decreased GABAergic Neurons, Autism-like Behavior, and Increased Oxidative Stress Cascades

**DOI:** 10.1007/s12035-018-1450-9

**Published:** 2019-01-11

**Authors:** Zhu Li, Rekha Jagadapillai, Evelyne Gozal, Gregory Barnes

**Affiliations:** 1 Department of Neurology, Vanderbilt University School of Medicine, Nashville, TN USA; 2 Department of Pediatrics, Vanderbilt University School of Medicine, Nashville, TN USA; 3 Department of Pediatrics, University of Louisville School of Medicine, Louisville, KY USA; 4 Department of Neurology, University of Louisville School of Medicine, Louisville, KY USA; 5 Pediatric Research Institute, University of Louisville Autism Center, 1405 East Burnett Ave, Louisville, KY 40217 USA

**Keywords:** Autism, Epilepsy, Semaphorin, Interneuron, Hippocampus, Development

## Abstract

Autism and epilepsy are diseases which have complex genetic inheritance. Genome-wide association and other genetic studies have implicated at least 500+ genes associated with the occurrence of autism spectrum disorders (ASD) including the human semaphorin 3F (Sema 3F) and neuropilin 2 (NRP2) genes. However, the genetic basis of the comorbid occurrence of autism and epilepsy is unknown. The aberrant development of GABAergic circuitry is a possible risk factor in autism and epilepsy. Molecular biological approaches were used to test the hypothesis that cell-specific genetic variation in mouse homologs affects the formation and function of GABAergic circuitry. The empirical analysis with mice homozygous null for one of these genes, Sema 3F, in GABAergic neurons substantiated these predictions. Notably, deletion of Sema 3F in interneurons but not excitatory neurons during early development decreased the number of interneurons/neurites and mRNAs for cell-specific GABAergic markers and increased epileptogenesis and autistic behaviors. Studies of interneuron cell-specific knockout of Sema 3F signaling suggest that deficient Sema 3F signaling may lead to neuroinflammation and oxidative stress. Further studies of mouse KO models of ASD genes such as Sema 3F or NRP2 may be informative to clinical phenotypes contributing to the pathogenesis in autism and epilepsy patients.

## Introduction

Autism spectrum disorder (ASD) is a neurobiologic disorder with deficits in social skills and language and the presence of restrictive interest/repetitive behaviors [1]. The best estimates for etiologies of ASD are about 50–90% genetic and 10–50% environmental causes [2–5]. In addition to the 500–1000 genes identified as risk for ASD, multiple environmental causes including maternal stress, diabetes, infection/inflammation, obesity, and medications have been implicated [6]. Additive factors such as terbutaline and maternal stress during gestation produce autistic-like behaviors, spontaneous recurrent convulsive seizures, epileptiform EEGs, and hippocampal gliosis in rodents similar to patients with autism and epilepsy [7]. Those gene–environment interactions may target cortical interneurons, predisposing to autism and epilepsy [8]. Functional genomic work has implicated a wide array of transcriptome pathways (synaptic, immune, cell cycle, DNA damage, WNT signaling, cortical patterning/differentiation in ASD) [9]. A recent report documented reductions of the axon guidance ligand semaphorin 3F (Sema 3F) and its receptor, neuropilin 2 (NRP2), expression in pregnant women with preeclampsia, another putative risk factor for ASD [10–12]. Thus, the interactions of pregnancy factors with developing brain can impact biological processes such as GABAergic neurogenesis, thereby contributing to the development of ASD.

Neurodevelopmental disorders such as ASD and epilepsy result from GABAergic dysfunction involving complex multifactoral etiologies including gene–environment interactions [13–15]. Molecular and genetic studies suggest that signaling pathways necessary for GABA interneuron development are disrupted in both ASD and epilepsy [16–22]. Indirect genetic evidence suggests that one particular signaling pathway, the semaphorin (Sema) pathway, is involved with both disorders [23, 24]. Sema 3F is known to interact with a number of ASD proteins including, the Fragile X protein, MECP2, the gene product in Rett syndrome, and neural cell adhesion molecule [25–27]. An interneuron phenotype recapitulated in mice homozygous null for neuropilin 2 (NRP2), the receptor of Sema 3F, is similar to neuropathology found in ASD brains [18]. Behavioral studies in the Sema 3F and NRP2 null mice recapitulate some aspects of autistic behaviors [28, 29]. Thus, mutants in neuropilin and semaphorin signaling are important mouse models for the understanding of neurodevelopmental disorders, especially those involving GABAergic dysfunction.

Guidance cues such as semaphorin–neuropilin signaling systems may be intimately involved in GABA-mediated processes early in development and, more recently, in oligodendrocyte development [30]. NRP2 and its ligand Sema 3F are known neuronal molecules that outside of the CNS regulate proliferation, migration, and differentiation of a variety of cell types including vascular, immune, renal, auditory, and cardiac cells [31]. Sema 3F–NRP2 signaling controls dendrite and spine development, excitatory synaptogenesis, long-term potentiation, and excitatory neural transmission during development of hippocampal principal neurons [32–35]. However, the role of Sema 3F–NRP2 signaling in the development of hippocampal and cortical GABAergic circuitry has been less well characterized. NRP2 signaling is involved in cell positioning of adult-born neurons through regulation of glycogen synthase kinase-3 [36]. NRP2 expression in precursors located in the ventral ventricular zone as well as tangentially migrating GABA+ interneurons is noted when these neurons enter the cortical plate [19, 21, 37]. Regarding migration, the NRP2 receptor ligand (Sema 3F) is expressed in the cortical plate and striatum during development along the cortical/hippocampal GABAergic neuron migratory pathway. Ectopic Sema 3F expression can alter GABAergic neuron migration [19, 21, 37]. The transcription factors DLX1, Nkx2.1, and COUP-TFII likely regulate the NRP2 promoter transcription, allowing some interneurons to enter the specific regions of the striatum, neocortex, and amygdala [20, 38–41]. Data from the NRP2 knockout mouse suggest that NRP2 signaling is necessary both for interneuron migration and differentiation and for the development of the postsynaptic GABAergic synaptic apparatus in pyramidal cells [18, 19, 21, 37, 42, 43].

The focus of this study was to determine during development what cell types express biological relevant Sema 3F, determine its role in epileptogenesis and behavior, and identify possible biochemical pathways to direct the migration and differentiation of interneurons. Increased inflammatory responses and oxidative stress impairing the migration, differentiation, and function of interneuron may be a common mechanism in neurodevelopmental disorders [44–46]. Further, reactive oxygen species may be important for semaphorin function including endosomal sorting and regulation of the cytoskeleton [47]. Pathogenic copy number variants as well as individual gene variants including the Sema 3F and NRP2 gene are associated with autism, epilepsy, intellectual disability, developmental delay, and dysmorphia in humans (NCBI-based ClinVar database 2017 [23, 24]). Utilizing molecular biological, anatomy, behavioral, and neurophysiological investigations, we confirmed that the predicted molecular components of Sema 3F signaling, including the increase in reactive oxygen species/neuroinflammation, are associated with variations in GABAergic circuitry. These observations suggest that similar anomalies in humans may contribute to neuropathology in ASD and epilepsy.

## Methods

### Generation of Semaphorin 3F Conditional Knockout Mice

The animal experiments were performed under protocols approved by the Vanderbilt University Institutional Animal Care and Use Committee. We received conditional semaphorin 3F heterozygotes (Sema 3F^F/+^; one of the two Sema 3F alleles was the floxed Sema 3F transgene) from Dr. Alex Kolodkin (Johns Hopkins University) on a C57BL/6J genetic background [33]. The DLX5/6^Cre^ heterozygotes (DLX5/6^Cre/+^, one of the two alleles was the DLX5/6^Cre^ transgene; referred to hereafter as DLX5/6^Cre^ targeting to GABAergic inhibitory neurons) from Jackson Laboratory are maintained on a mixed FVB/NJ and C57BL/6J genetic background [48, 49]. The EMX1^Cre^ heterozygotes from Jackson Laboratory (EMX1^Cre/+^, one of the two alleles was the EMX1^Cre^ transgene; referred to hereafter as EMX1^Cre^ targeting to excitatory neurons) are maintained on a C57BL/6J genetic background [50]. The animals were backcrossed five generations; each time, a heterozygous male was mated with a pure C57/BL/6J wild-type female (Jackson Laboratory colony). Cre heterozygotes-Sema 3F^F/+^ were mated together to produce homozygous Cre-Sema 3F^F/F^ mice. Genotypes were obtained at birth in the appropriate proportions. After obtaining tail DNA, PCR genotyping of each mouse was performed according to previously published protocols [18]. Handling-induced seizures, status epilepticus, and/or death were noted only in mice with either DLX5/6^Cre^-Sema 3F^F/+^ or DLX5/6^Cre^-Sema 3F^F/F^ genotypes. No epileptic events were noted among the EMX1^Cre^-Sema 3F^F/F^ mice. RT-PCR was performed as previously described [18].

### Cell Counts of Pyramidal Cells and Interneurons

At the end of the behavioral testing, the animals were anesthetized with isoflurane and perfused with 4% PFA, and brains were collected for immunohistochemistry and cell counts. Frozen tissue was cut with a freezing microtome to prepare 20-μm-thick slices. Different cell types and interneuron populations were identified by specific markers immunostaining as delineated below. Using an Olympus BX61 microscope and ×40 objective (N.A. 0.75), cell count analyses of pyramidal and interneuron cell numbers in CA3/hilar regions, other hippocampal subfields, and other brain regions were accomplished as modified from a previous modified semiquantitative method using the Neurolucida system [18]. To avoid potential bias, the experimenter was blinded to conditions and genotype during all histological procedures. The region of interest in terms of volume was defined from 5 matching 50 μm coronal sections (200 μm part) per mouse between the septum (bregma level + 0.75 mm) and through the extent of the dorsal hippocampus − 2.5 mm). Potential changes in the size of nuclei and hippocampal volume and change in section thickness were considered but found either not significant (nuclei, hippocampal volume) or changed uniformly among all sections (~ 50% decrease in section thickness after processing). The top of the nuclei or soma of excitatory and GABAergic cells were counted in the cell layers of three major hippocampal areas; dentate gyrus/hilar region, CA3, and CA1 regions; and in other brain regions only if the whole nuclei was within the region of interest [18, 51]. Nissl stain, a Cresyl violet acetate solution, uses basic aniline dye to stain RNA blue and is used to highlight important structural features of neurons in paraformaldehyde or formalin-fixed tissue. Nissl or immunocytochemical cell counts were counted through the entire thickness of the section and quantified as cell densities. Each leaf (right and left hemispheric brain regions) was quantified and averaged as a cell density measurement per section in a given brain area. The cell density per section was calculated for a given region in each of the five sections that were averaged together to establish a single value for cell density per region for each mouse. Immunocytochemistry was performed as previously described [18, 51]. For labeling the various types of GABAergic interneurons, GABA, GAD-67, parvalbumin, calretinin, somatostatin, and neuropeptide Y cell counts, the *total* number of cells counted in each hippocampal subregion was noted as well as the cell density. The averages per region were calculated from the brains from at least five animals per genotype. We made the assumption that antibody penetration and stain penetration were uniform among the sections. Abercrombie’s formula for the ratio of “real” cell numbers to observed cell numbers to correct for overestimation was applied to each data point (in this case for 50 μm section and cell of 10 μm in diameter—0.83). The statistical analysis was performed using ANOVA post hoc Tukey’s multiple comparison test analysis. Similar analyses were done for both pyramidal and interneuron cell counts among all genotypes.

For counting of GABAergic synapses, sections (at 200 μm intervals) per mouse/genotype were obtained and counted using the Image-Pro Plus program as previously described [18]. Confocal microscopy [Zeiss Laser Confocal Scanning Microscope (LSM); Carl Zeiss] images of 0.5 μm sections (Z-series “stacks”) were taken along the *Z* axis in the CA3b region using the ×100 oil immersion objective. The images taken from different sections were compared under constant conditions by adjusting the LSM parameters (pinhole size, laser power, detector voltage). Punctata below 0.1 μm^2^ were eliminated. Cells were counted only if the clearly defined largest diameter of the cell and clearly defined punctata could be visualized. The CA3b regions of both the left and right hippocampi were counted. The number of Parv+ and GAD-65+ punctata surrounding 50 pyramidal cells was counted as well as the percent double labeling with both markers. Experimenters were blinded to genotype and treatment group, and average measures per animal were used for statistical analysis (*n* = 5/group). Statistical analysis was performed using ANOVA with post hoc Tukey’s multiple comparison test analysis.

### Quantitative Analysis of Synaptosomal Proteins on Immunoblots

Synaptosomes were prepared from adult mice (>P60, *n* = 3 per group) as previously described [18]. In some experiments, synaptosomes (10 μg protein) were diluted in RIPA buffer followed by the addition of SDS gel sample buffer and analyzed as follows. Briefly, 10 μg of hippocampal synaptosomal protein was applied to SDS-PAGE gels followed by transfer to Immobilon P membranes (Millipore, Billerica, MA). After blocking membranes with 1× PBS, 0.1% Tween-20, and 5% milk, proteins were visualized by primary antibody [1:4000 NRP2 antiserum, gift of Alex Kolodkin, 1:1000 NeuN from AbCam, 1:1000 semaphorin 3F from AbCam], followed by secondary antibody detection with horseradish peroxidase (HRP)-linked anti-rabbit or anti-mouse IgG (1:5000; Jackson ImmunoResearch) using Supersignal West Pico Chemiluminescent Substrate (Pierce, Rockford, IL, USA). Light emission was detected by BioMax film (Sigma-Aldrich, St Louis, MO). Optic densities (OD) of detected bands were expressed as a mean of the ratios of experimental versus wild-type mouse values ± SEM. Statistical analysis was performed using ANOVA with post hoc Tukey’s multiple comparison test analysis.

### Monitoring of Epileptogenesis and Behavioral Studies

All behavioral and EEG experiments were conducted in the Vanderbilt Murine Neurobehavioral core. Animals were acclimated to the core environment for at least 7 days prior to testing. Animals were housed on a 12-h light–dark cycle with free access to food and water. All animals were tested at ages between P60 to P180. Prior to all testing, animals were acclimated for 20 min in an anteroom of the primary observation room.

#### Pentylenetetrazol Seizure Threshold

Pentylenetetrazol (PTZ) is a blocker of GABA receptor and can be easily used to quantify seizure susceptibility. In particular, PTZ kindling is a quantitative paradigm which can distinguish seizure susceptibility among genotypes. PTZ was dissolved in sterile 1× PBS and administered SC at a dose of 50 mg/kg. This dose was used because 75% of mice with C57BL/6J background had convulsive activity at this dose. Animals were monitored for 45 min after injection. Mice that did not develop seizures during the observation period were excluded from analyses. Behavioral seizures were scored on the following scale: class 1, hypoactivity; class 2, focal clonus (clonic seizure activity affecting the head, face, or forelimbs); class 3, generalized clonus (sudden loss of upright posture, whole body clonus involving all four limbs and tail, rearing, and autonomic signs); and class 4, tonic–clonic seizures (generalized seizure characterized by tonic limb extension) [52]. Latency to the first seizure as well as seizure class was recorded. The statistical analysis was performed using ANOVA with post hoc Tukey’s multiple comparison test analysis.

*Continuous video EEG* was performed on DLX5/6^Cre^-Sema 3F^F/F^ KO and littermate control mice (*N* = 4–6/genotype). Prefabricated EEG head mounts (Pinnacle Technologies, Lawrence, KS, USA) were surgically implanted. EEG recordings were performed in adjacent recording chambers. Mice were allowed free access to food and water. A 12-h light–dark cycle was maintained for the duration of the recording. Twenty-four hours of VEEG recording of two-channel EEG data was reviewed for each mouse which were representative of the rostral and caudal brain regions. Simultaneous video and electromyography were available to confirm the sleep and awake cycles. Visual analyses and EEG spectral analyses of spike wave, slowing, and EEG power were performed. The first 10 min of each hour of waking EEG was chosen for performance of absolute power values (uV2) on the delta (0.5–4 Hz), theta (4–8 Hz), alpha (8–12 Hz), and beta (13–30 Hz) frequency bands. The values obtained were averaged over the 12-h day of each 24 h VEEG recording session. To account for differences in the absolute power between mice, the mean power at each frequency was normalized to the total power of all frequencies for each mouse.

#### Social Preference and Social Novelty Testing

The three-chamber social interaction test [53] was performed to measure social behavior. Weight-matched mice of the same genotype which had been housed in different cages were placed in the middle chamber and allowed to explore freely for 10 min. Then, the doors to chambers were opened to allow the mouse to explore side chambers containing either an unfamiliar or familiar mouse housed in a steel cage or an inanimate object in a cage. The number of contacts, total duration of each contact, total distance traveled, and total duration in each chamber were recorded and automatically analyzed, but results were confirmed via manual analyses of videotape. Statistical analysis was performed using ANOVA with post hoc Dunn’s multiple comparison test analysis.

#### Home Cage Monitoring

To evaluate for the possibility of increased repetitive behaviors, DLX5/6^Cre^-Sema 3F^F/F^ KO and wild-type littermate mice (*N* = 8–14/group) were video-recorded alone in their home cage for 24 h while maintaining their light–dark schedule as previously described [54]. Automated video analyses were conducted using HomeCage Scan (CleverSys, Inc., Reston, VA, USA) to index the time spent performing individual behaviors. The resulting data was condensed into 10 individual behaviors: distance traveled, eat/drink, hang, jump, rear, walk, groom, sleep/awake, and total behaviors. The total behavior time was not significantly different among the four genotypes. Bouts of hanging were defined as distinct periods of hanging from the wire cage lid separated by unique nonhanging behaviors. Statistical analysis was performed using ANOVA with post hoc Dunn’s multiple comparison test analysis.

#### Open Field Testing

Exploratory locomotor activity was explored and evaluated using the BLANK system and software. Exploratory locomotor activity was measured in a commercially available open field arena (ENV-520, MED Associates, Georgia, VT) measuring 27 × 27 cm, over a period ranging from 5 min to 24 h. Infrared beams and detectors automatically detect and record the subject’s *X*–*Y*–*Z* coordinates every 50 ms. Post-Session software analyses can provide the distance traveled, rearings, circling behavior, repetitive or stereotypical behaviors, etc. Statistical analysis was performed using ANOVA with post hoc Dunn’s multiple comparison test analysis.

#### Marble Burying Test

Mice are placed in individual cages containing ~ 5 cm of beta chip sawdust bedding for 15 min to acclimate to the test conditions. After 15 min, each mouse is briefly removed from its cage, and standard glass toy marbles (assorted styles and colors, 15 mm diameter) were placed gently on the surface of the bedding in five rows of four marbles. The mice are returned to the cage and allowed to remain in the cage undisturbed for 30 min. At the end of the study, the mice are returned to their home cages and the number of marbles buried in the bedding is recorded [55]. Statistical analysis was performed using ANOVA with post hoc Dunn’s multiple comparison test analysis.

### Immunofluorescence of Oxidative Stress and Neuroinflammation

Immunohistochemical studies were carried out to assess oxidative stress and inflammation in the cortex, hippocampus, and amygdala of DLX56/6^Cre^-Sema 3F^F/F^ KO mice (Cre+/FF), compared to control mice (Cre−/FF, Cre+/WT, Cre−/WT) lacking either Cre expression or flox sites flanking the Sema 3F gene, or both, and therefore, expressing normal levels of Sema 3F. Five animals per group were used to determine Iba1 expression (for microglia), 4-hydroxy-2-nonenal (4-HNE, for lipid peroxidation), and dihydroethidium (DHE, for superoxide) in each brain region. Mice were anesthetized and perfused transcardially by 4% paraformaldehyde. The brains were postfixed; cryoprotected sequentially in 15, 30, and 40% sucrose; and embedded in tissue freezing medium for cryosectioning in 30-μm-thick sagittal sections using a cryostat (Leica, Germany) and then mounted onto charged slides.

Red fluorescent DHE staining was obtained by incubating the slides in 20 μmol/L DHE (Sigma Chemical Co, St Louis, MO) for 30 min. The sections for immunofluorescence were blocked with normal donkey serum and incubated overnight at 4 °C with primary antibodies against Iba1 (1:500; Wako Pure Chemical Industries, Ltd., Japan) and 4-HNE (1:50; JaICA, Japan). The sections were incubated with FITC-tagged anti-rabbit (for Iba1) or TRITC-tagged anti-mouse (for 4-HNE) secondary antibodies (Jackson ImmunoResearch, West Grove, PA) for 1 h at room temperature in the dark and then mounted, and images were captured using a laser scanning confocal microscope (Leica-TCS SL, Wetzlar, Germany). As negative controls, sections were preincubated with superoxide dismutase (SOD; 500 U/mL) before DHE staining, or with rabbit IgG or mouse IgG in lieu of the antibody for Iba1 and 4-HNE staining, respectively. These negative controls were used to set the baseline threshold for fluorescence quantifications.

Images were stored and quantified by image analysis software (Image-Pro Plus). Data were expressed as intensity per unit area and analyzed by ANOVA, followed by post hoc Tukey’s multiple comparison test using statistical software (GraphPad Prism, La Jolla, CA).

## Results

### Cell Subtype Deletion of Semaphorin 3F and Verification of Semaphorin 3F Recombination

**Fig. 1:**
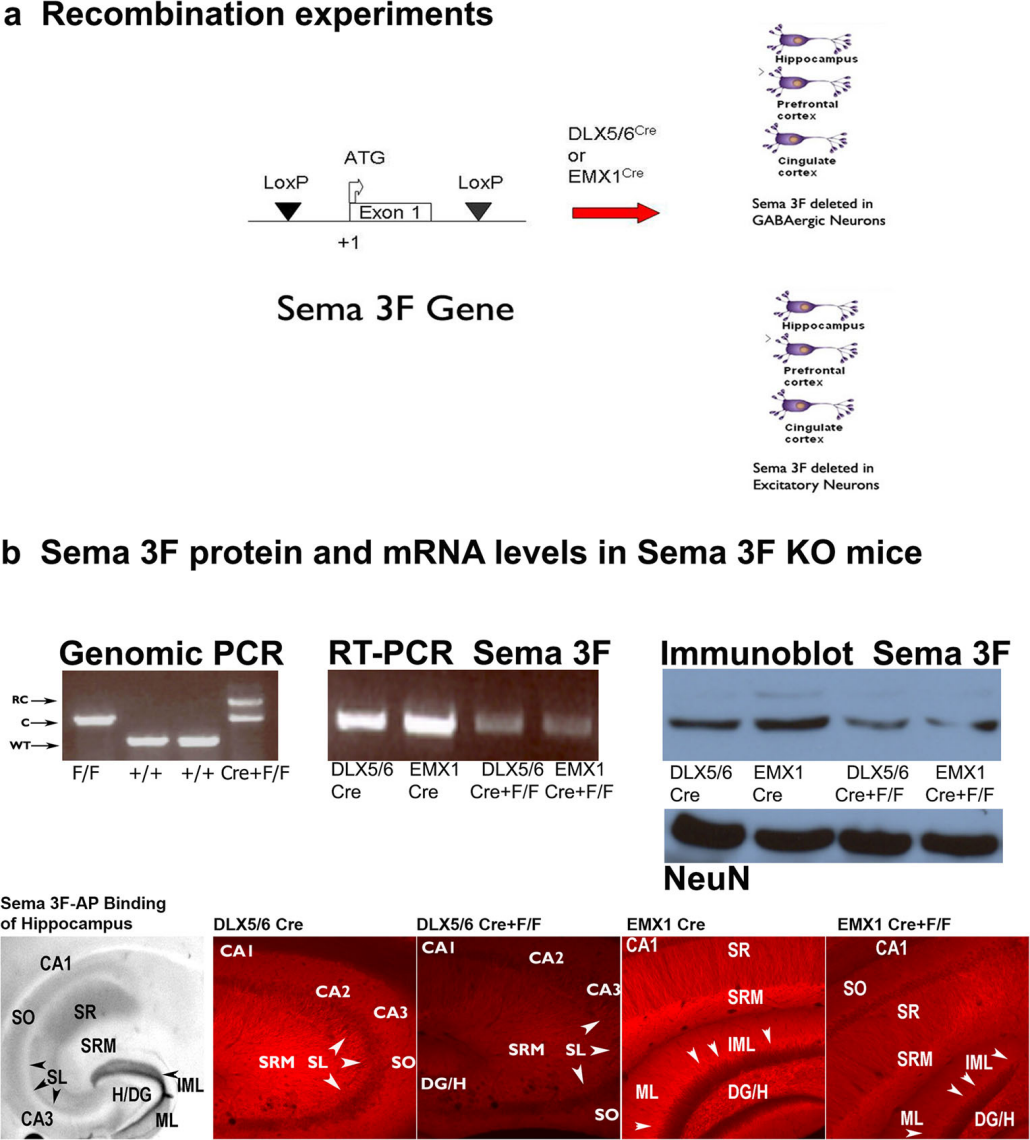
Expression of semaphorin 3F in GABAergic and excitatory cell pathways. In **a**, the Cre recombinase is expressed in either postmitotic interneurons or pyramidal cells. Postmitotic neurons have the ligand for neuropilin 2 (NRP2) and semaphorin 3F (Sema 3F), deleted in a cell-specific manner. In **b**, the genomic PCR shows that the recombined allele is only partial in either cell-specific knockout mouse. RC = recombined allele, C = conditional allele, WT = wild-type allele. Correspondingly, the hippocampal homogenates of (+Cre F/F) mice contained 55% less Sema 3F protein compared to (+/+) mice. Tests of hippocampal DNA (lane 1, Sema 3F^F/F^; lane 2, +/+; lane 3, +/+; lane 4, DLX5/6^Cre^-Sema 3F^F/F^), protein, and total cellular RNA suggest partial recombination results in a reduction in Sema 3F production but not NeuN, a marker of total neuron numbers. The Sema 3F-AP binding demonstrates the localization of the NRP2 in dendritic regions of the hippocampus. The semaphorin 3F ICC demonstrates the same localization and reductions of Sema 3F in both DLX5/6^Cre^-Sema 3F^F/F^ (deletion in GABAergic neurons) and EMX1^Cre^-Sema3F^F/F^ (deletion in excitatory neurons) mouse hippocampal dendritic regions. Each cell-specific knockout mouse is a hypomorph where the other neuronal types and glia still express Sema 3F. Abbreviations: *+/+* = wt for Sema 3F allele, no Cre; *EMX1*^*Cre*^*or DLX5/6*^*Cre*^ = Cre present in excitatory or GABAergic neurons, wt type Sema 3F allele; *Sema3F*^*F/F*^ = both Sema 3F allele are floxed, no Cre. The DLX5/6^Cre^-Sema 3F^F/F^ (full Sema 3F deletion in GABAergic neurons: *DLX5/6*^*Cre+*^*F/F*) and EMX1^Cre^-Sema3F^F/F^ (full Sema 3F deletion in excitatory neurons: *EMX1*^*Cre+*^*F/F*) are the cell-specific knockout mouse lines

**Fig. 2:**
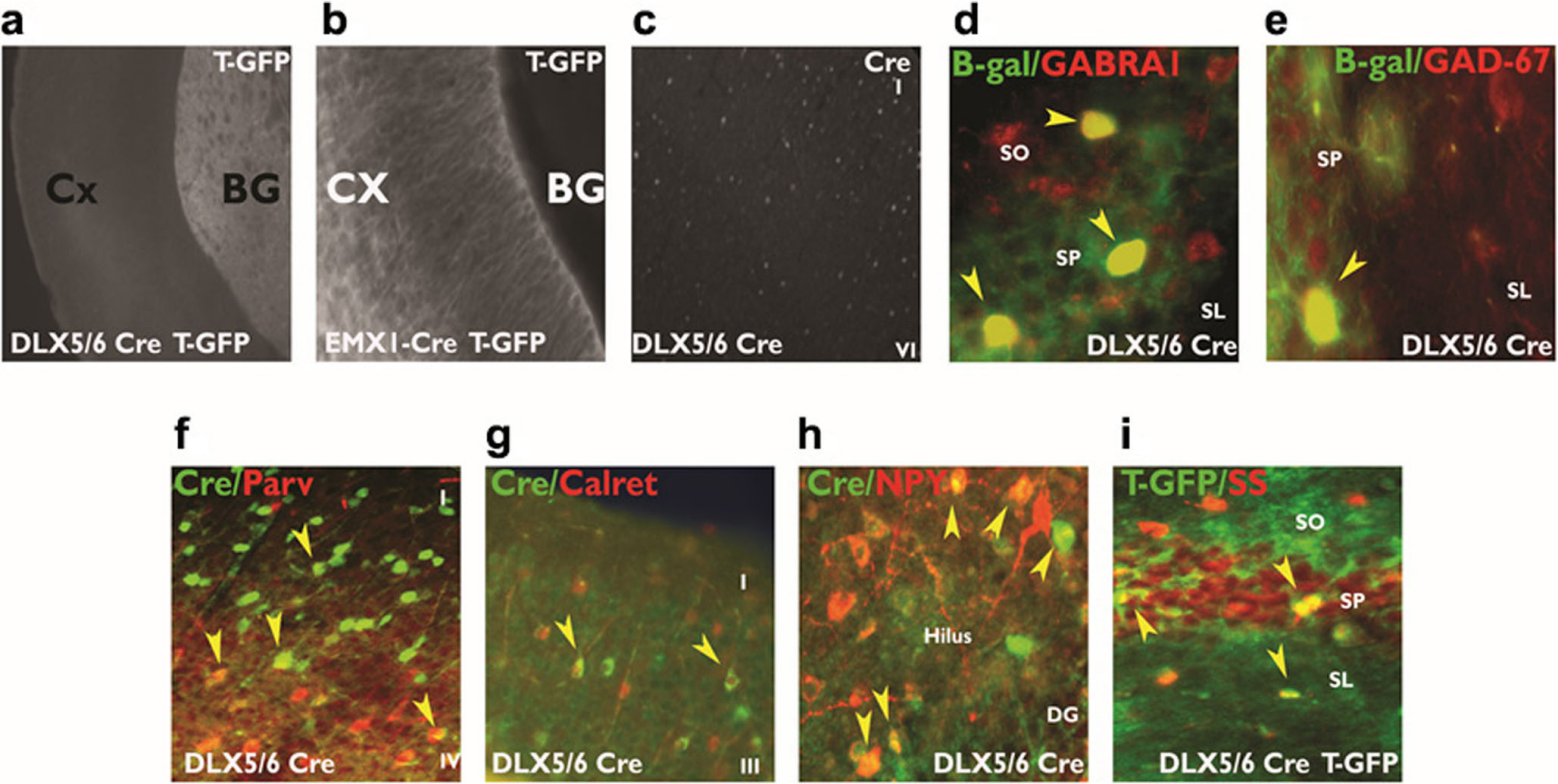
Recombination experiments in excitatory and inhibitory neurons. DLX5/6^Cre^ and EMXI^Cre^ driver genes were recombined in mice with the tomato-GFP (T-GFP) or beta-galactosidase (β-Gal) genes. T-GFP was expressed either **a** in ventral (DLX5/6^Cre^) telencephalic regions (basal ganglia) or in **b** dorsal (EMX1^Cre^) telencephalic regions (cortex) upon recombination. Cre protein immunocytochemistry (ICC) was also performed (**c**). Recombined β-Gal protein was expressed in hippocampal cells marked with GABAergic markers for the GABA_A_ receptor α1 (**d**) subunit of GAD-67 (**e**). With ICC, Cre protein was expressed in the cortex or hippocampus within parvalbumin+ (**f**), calretinin+ (**g**), neuropeptide Y+ (**h**), and somatostatin+ (**i**) interneuron subpopulations

Our intent using the Cre/loxP was to determine if there was a putative autocrine or paracrine link between semaphorin 3F signaling, GABAergic differentiation, and cell type. We utilized two mouse strains: one strain (DLX5/6^Cre^-Sema 3F^F/F^) with a developmentally timed conditional deletion of the Sema 3F gene in postmitotic immature GABAergic neurons or another strain (EMX1^Cre^-Sema 3F^F/F^) with a developmentally time conditional deletion of the Sema 3F gene in excitatory neurons (Fig. 1). The conditional mice progeny displayed recombination of the conditional Sema 3F gene and a 53–90% reduction of Sema 3F mRNA/protein levels only in the hippocampus of mice with DLX5/6^Cre^ and EMX1^Cre^ transgene expression (Fig. 1) [48, 49]. Binding of Sema 3F-AP is present in all neurite layers of the murine hippocampus but especially high density in CA3 stratum lucidum and inner molecular layer of the dentate gyrus (Fig. 1). Expression of the Sema 3F protein is detected in these same hippocampal regions but is again decreased by deletion of the Sema 3F gene from either interneurons or pyramidal cells (Fig. 1). Crossing the DLX5/6^Cre^ or EMX1 X^Cre^ mice with beta-galactosidase ROSA26 reporter mice or tomato-GFP mice show that DLX5/6^Cre^ or EMX1^Cre^-mediated recombination only occurs in the hippocampal interneurons or excitatory neurons as detailed by excitatory or GABAergic markers double labeled with the Cre recombinase or beta-galactosidase. These data suggest recombination occurred in ventral or dorsal telencephalic neurons during development, respectively (Fig. 2).

### Interneuron-Derived Semaphorin 3F Regulates Neural Excitability

**Fig. 3:**
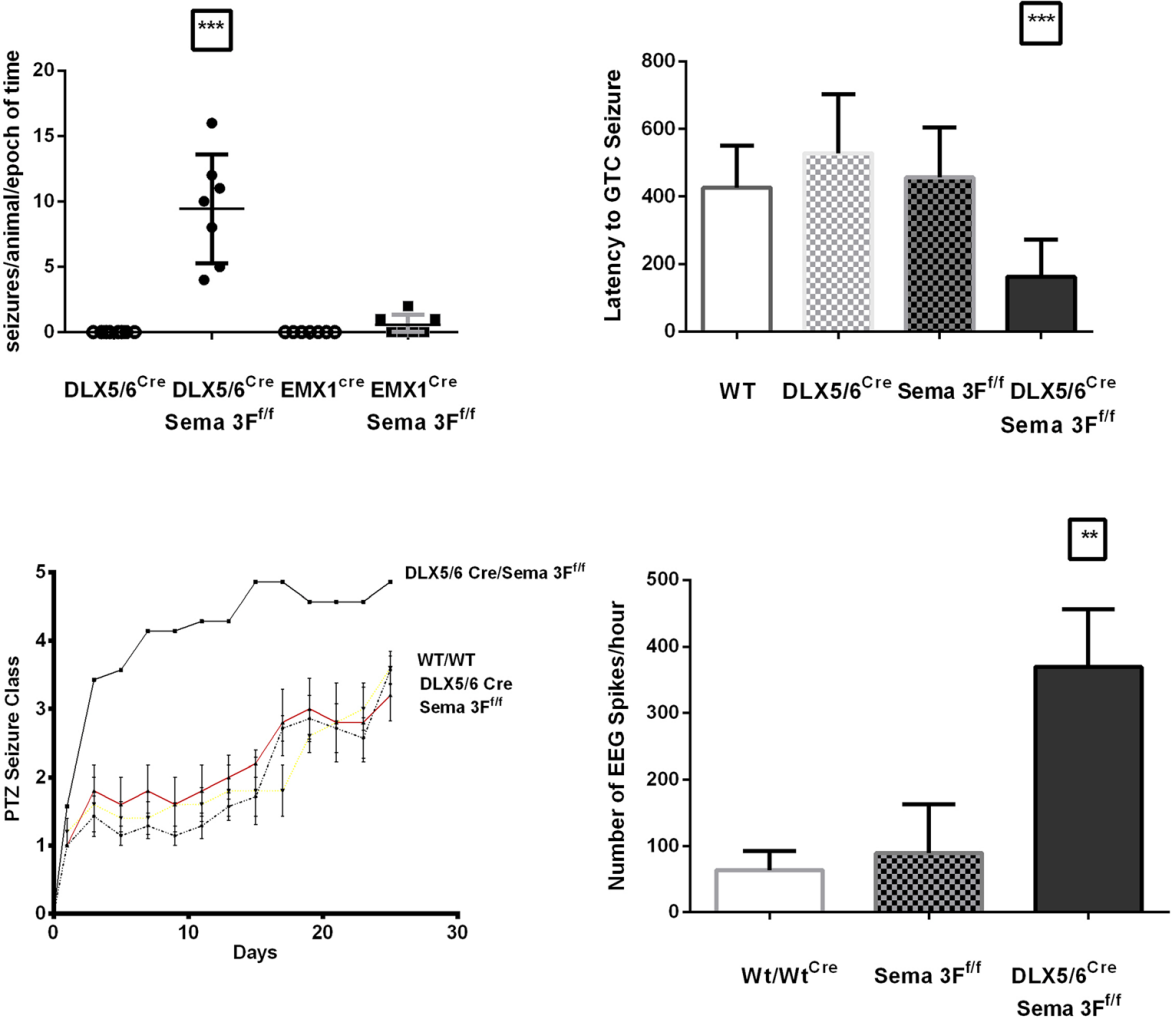
Epileptogenesis in semaphorin 3F cell-specific mutant mice. Video EEG experiments captured both clinical and electrographic recordings of epileptic seizures only the DLX5/6^Cre^-Sema 3F^F/F^ but not EMX1^Cre^-Sema 3F^F/F^ mice (top left panel). Use of a GABA_A_ receptor blocker pentylenetetrazole (PTZ) resulted a much shorter latency to first GTC seizure in single convulsant injection of DLX5/6^Cre^-Sema 3F^F/F^ mice compared to other genotypes (top right panel). Through the use of subconvulsant doses in the PTZ kindling model, DLX5/6^Cre^-Sema 3F^F/F^ mice reached three GTC seizures in a much shorter period compared to other genotypes (bottom left panel). Finally, the cortical EEG of DLX5/6^Cre^-Sema 3F^F/F^ mice had significantly more > 300 μV spike wave discharges versus the other genotypes (bottom right panel)

Given previous data from the NRP2 KO mouse [18], we investigated whether the source of Sema 3F from interneurons or pyramidal cells affected neural excitability. The physiological relevance of Sema 3F regulation of GABAergic synaptic function is underscored by the decreased latency (Fig. 3) to PTZ-induced generalized tonic–clonic seizures (DLX5/6^Cre^-Sema 3F^F/F^ 125 ± 15 vs. wild type 400 ± 50, *p* < 0.03) in DLX5/6^Cre^-Sema 3F^F/F^ mice. Similar latencies to PTZ-induced generalized tonic–clonic seizures (EMX1^Cre^-Sema 3F^F/F^ 372 ± 37 vs. wild type 392 ± 45) were observed in EMX1^Cre^-Sema 3F^F/F^ and wild-type mice (data not shown). We administered a subconvulsant 30-mg/kg PTZ every other day for 30 days to investigate epileptogenesis in the Sema 3F interneuron-specific knockout mice. The data indicated that DLX5/6^Cre^-Sema 3F^F/F^ mice reached three class V (or generalized tonic–clonic) seizures 10 days or more ahead of littermates with other gentoypes (*p* < 0.0001, Fig. 3). Semaphorin 3C (Sema 3C) is another ligand at the neuropilin 2 receptor. In contrast, the latency or PTZ kindling was not significantly different in Sema 3C KO heterozygotes compared to wild littermates (data not shown). Additionally, long-term video EEG revealed electrographic and clinical seizures only in the DLX5/6^Cre^-Sema 3F^F/F^ mice but not other genotypes (Fig. 3). The spike count per unit of time was only increased significantly in DLX5/6^Cre^-Sema 3F^F/F^ mice but not other genotypes (Fig. 3).

### Loss of Interneuron-Derived Semaphorin 3F Decreases Subpopulation Numbers of GABAergic Neurons in Adult Mice

**Fig. 4:**
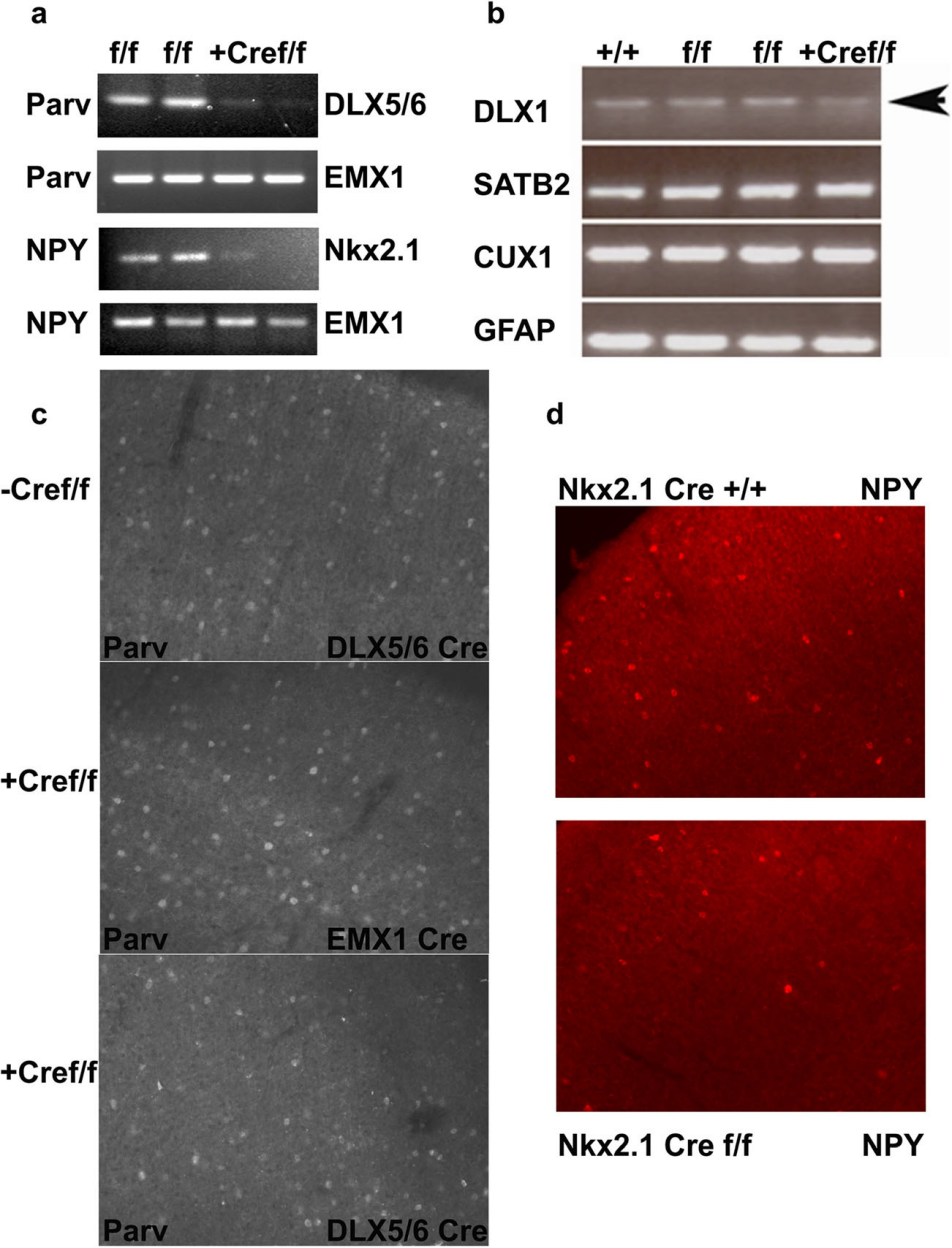
Decreased GABAergic markers in DLX5/6^Cre^-Sema 3F^F/F^ and Nkx2.1^Cre^ Sema 3F^F/F^ Mice. Sections from WT (+/+), Sema 3F^F/F^, DLX5/6^Cre^, EMX1^Cre^, EMX1^Cre^-Sema 3F^F/F^, DLX5/6^Cre^-Sema 3F^F/F^, Nkx2.1^Cre^, or Nkx2.1^Cre^ Sema 3F^F/F^ (*N* = 7 for each group) mice were subjected to immunocytochemistry or RT-PCR analyses (**a**, **d**). Decreased parvalbumin RNA or Parv+ cells were seen in the cortex of DLX5/6^Cre^-Sema 3F^F/F^ mice compared to other genotypes (**a**, **c**). DLX1 RNA, a marker of mature postmitotic interneurons, was decreased in hippocampus of DLX5/6^Cre^-Sema 3F^F/F^ mice compared to other genotypes (**b**). Glial (GFAP) and pyramidal cell (SATB2 and CUX1) markers were unchanged in all genotypes (**b**). In an alternative experiment, the use of a Nkx2.1^Cre^ driver to deleted Sema 3F from postmitotic interneurons resulted in decreased neuropeptide Y (NPY+) RNA and decreased number of NPY+ cells in cortex of Nkx2.1^Cre^ Sema3F^F/F^ mice compared to other genotypes (**d**)

**Fig. 5:**
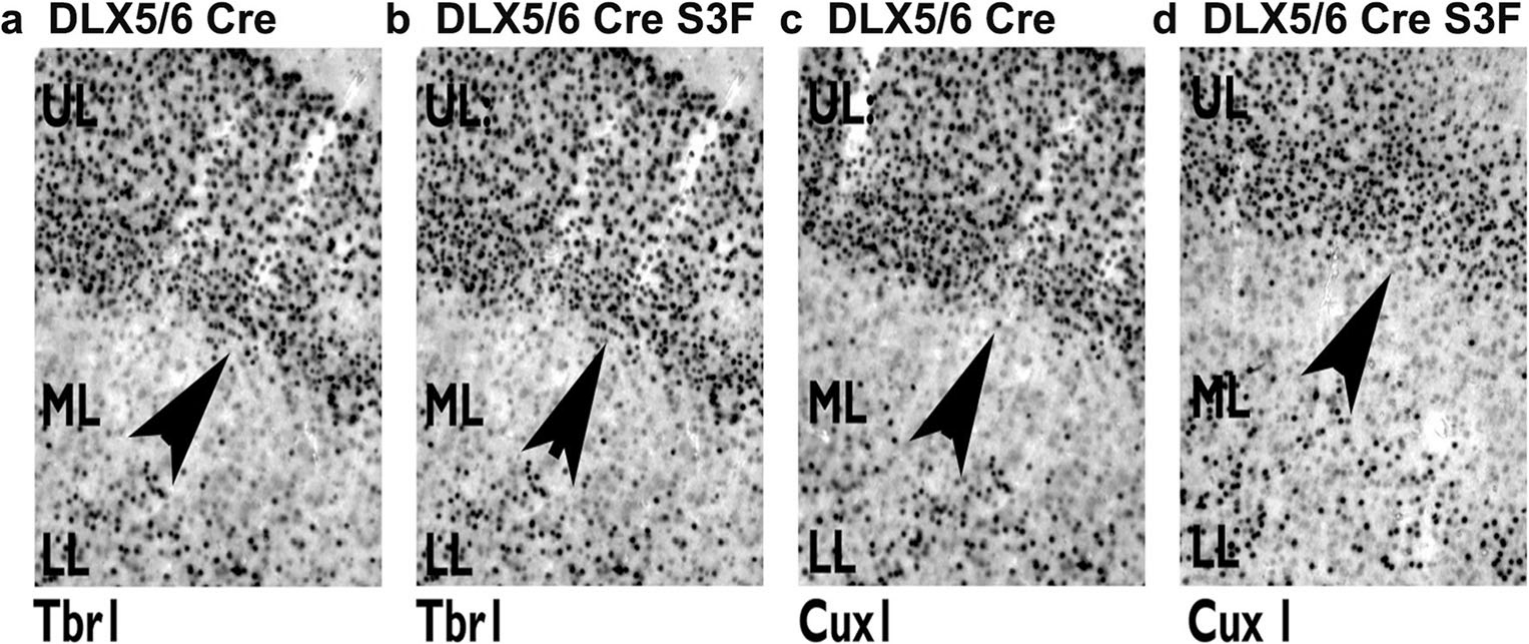
**a**–**d** No changes in density of glutamatergic cells with neocortex. Immunocytochemistry shows the unchanged density of Cux1+ and Tbr1+ cells in the cortex of either genotype (DLX5/6^Cre^, DLX5/6^Cre^-Sema 3F^F/F^ mice). These data are similar to cortical RNA findings (Fig. 4) and Nissl cell counts of hippocampal pyramidal cell layers (Table 3)

The decreased cell numbers of interneurons in NRP2 or Sema 3F constitutively knockout mice suggest that Sema 3F signaling via autocrine or paracrine processes could control migration, cell fate, or survival [42]. Thus, we examined the microanatomy of the hippocampus, cortex, and other brain regions via analyses of the major cellular populations in the adult mouse. Analyses of GABA+ neurons suggest a major decrease in cell numbers of the cortex and hippocampus in the DLX5/6^Cre^-Sema 3F^F/F^ and less so in EMX1^Cre^-Sema 3F^F/F^ mice (Table 1). Nissl stain, commonly used to identify the neuronal structure in brain and neural tissue, showed no differences in the number of Nissl-positive cells in each hippocampal pyramidal cell layers but 40–53% reduction in Nissl-positive cells with hippocampal dendritic layers (*p* < 0.05 to 0.001, Table 2) by ANOVA (*p* < 0.0001). Additionally, we counted the number of immunoreactive cells in sections for GABAergic markers (SS+, CR+, Parv+, NPY+, GABA+). There was a significant reduction in the total number of GABA+ (70%) and calretinin+ (CalR+) neurons (40%) in the mutant CA1 region of the hippocampus of DLX5/6^Cre^-Sema 3F^F/F^ but not EMX1^Cre^-Sema 3F^F/F^ mice (Table 1, Fig. 4) which explains the decrease in transcripts of the GABAergic marker DLX1 compared to glutamatergic (SATB2, CUX1) and glial (GFAP) markers (Fig. 4). In fact, deletion of Sema 3F by Nkx2.1-Cre resulted in decreased NPY+ neurons in the mutant cortex (Fig. 4). Analyses of Parv, CalR+, NPY, and SS+ neurons suggest that cell numbers are similar in many brain regions of DLX5/6^Cre^-Sema 3F^F/F^ mice, EMX1^Cre^-Sema 3F^F/F^ mice, and wild-type littermates (Table 1). Similarly, the principal cell numbers in Nissl-stained cell layers and markers of principal cells in cortex (Cux1 and Tbr1) were not significantly different among genotypes (Fig. 5). However, the somatosensory cortex showed similar 29 and 38% reduction in GABA+ cells and Parv+ cells within both the EMX1^Cre^-Sema 3F^F/F^ and the DLX5/6^Cre^-Sema 3F^F/F^ mice, respectively (Table 1, Fig. 4, *p* < 0.05 to 0.001).

**Table 1:** Cell number counts in excitatory and inhibitory neuron semaphorin 3F mutants

	EMX1^Cre^	EMX1 KO	*p* value^a^	DLX5/6^Cre^	DLX5/6 KO	*p* value^a^
GABA						
CA1	874 ± 62	770 ± 74	NS	826 ± 50	306 ± 95	*p* < 0.05
CA3	978 ± 62	644 ± 44	NS	881 ± 86	466 ± 81	NS
DG	666 ± 82	682 ± 88	NS	848 ± 65	416 ± 81	NS
SS	1895 ± 229	1354 ± 221	*p* < 0.05	1954 ± 66	1872 ± 302	NS
Parv+						
CA1	395 ± 48	408 ± 31	NS	483 ± 30	171 ± 15	NS
CA3	617 ± 69	645 ± 77	NS	619 ± 43	255 ± 26	NS
DG	257 ± 12	244 ± 21	NS	314 ± 21	55 ± 9	NS
SS	2134 ± 200	2073 ± 131	NS	2346 ± 194	1457± 202	*p* < 0.001
PF	1260 ± 124	1436 ± 74	NS	1477± 285	1460 ± 82	NS
BG	1033 ± 50	774 ± 49	NS	712 ± 128	899 ± 44	NS
NPY+						
CA1	407 ± 39	347 ± 82	NS	582 ± 30	308 ± 48	NS
CA3	410 ± 22	368 ± 62	NS	491 ± 57	427 ± 35	NS
DG	488 ± 50	480 ± 124	NS	588 ± 34	532 ± 47	NS
SS	1182 ± 108	887± 93	NS	1157 ± 112	849 ± 109	NS
PF	681 ± 87	560 ± 31	NS	740 ± 114	320 ± 32	NS
BG	546 ± 48	638 ± 54	NS	676 ± 57	610 ± 62	NS
CalR+						
CA1	971 ± 30	1024 ± 30	NS	985 ± 42	597 ± 64	*p* < 0.05
CA3	582 ± 43	666 ± 101	NS	642 ± 46	489 ± 76	NS
DG	1422 ± 92	1520 ± 88	NS	1476 ± 75	1115 ± 163	NS
SS	1624± 43	1906 ± 56	NS	1845 ± 99	1468 ± 152	NS

^a^*p* value significant by one-way ANOVA followed post hoc Tukey’s *t* test for multiple comparisons

*EMX1 KO* = EMX1^Cre^-Sema 3F^F/F^ mice; *DLX5/6 KO* = DLX5/6^Cre^-Sema 3F^F/F^ mice

**Table 2:** Nissl cell number counts in hippocampal subregions of semaphorin 3F mutants

	DLX5/6^Cre^	DLX5/6 KO	*p* value^a^	DLX5/6^Cre^	DLX5/6 KO	*p* value^a^
Dendritic regions*						
CA1 SR	71 ± 4	34 ± 1	*p* < 0.0001			
CA1 SO	48 ± 2	27 ± 4	*p* < 0.05			
CA3 SR	67 ± 4	61 ± 3	NS			
CA3 SO	58 ± 7	35 ± 2	*p* < 0.01			
Hilus	66 ± 7	36 ± 1	*p* < 0.0001			
Pyramidal cell layer*						
CA1				115 ± 14	109 ± 19	NS
CA3				123 ± 17	113 ± 22	NS
Hilus				185 ± 25	174 ± 32	NS

^a^*p* value significant by one-way ANOVA followed post hoc Tukey’s *t* test for multiple comparisons

*Cell counts were performed in five sections from each of five mice per genotype. The values are an average cell density per 200 μm^2^ as described in the “Methods” section

### Change in Cell-Specific Semaphorin 3F Expression Reduces GABAergic Markers of Murine Hippocampus

**Fig. 6:**
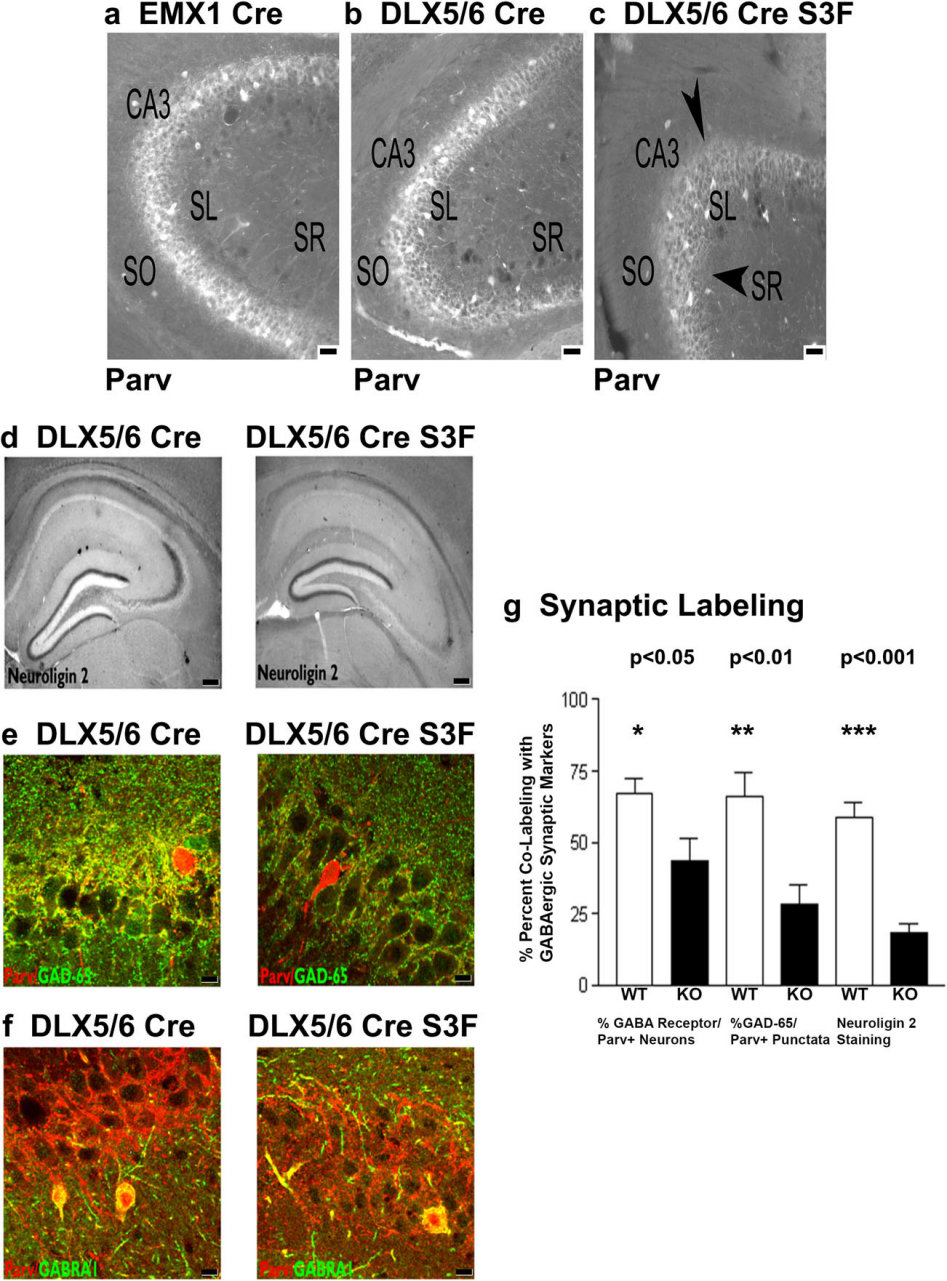
Semaphorin 3F expression regulates GABAergic neurite outgrowth and synapse numbers. Twenty micron sections from DLX5/6^Cre^, EMX1^Cre^, EMX1^Cre^-Sema 3F^F/F^, and DLX5/6^Cre^-Sema 3F^F/F^ (*N* = 7 for each group) mice were subjected to immunocytochemistry (**a**–**f**). Cell counts of (**a**–**c**) Parv+ neurons in the CA3 region were decreased in a nonsignificant trend (Table 1) in DLX5/6^Cre^-Sema 3F^F/F^ mice, but neurite staining and Neuroligin 2 staining of GABAergic synapses were significantly decreased in these same mice (**c**, **d**). As a confirmatory experiment, the number of GABAergic synapses (Parv+/GAD-65, yellow punctata) and GABA_A_ receptor α1+ interneurons (yellow label cells) is decreased 32 and 52%, respectively (**f**, **g**) (*p* < 0.0019 and *p* < 0.0001) in 20 μm sections from DLX5/6^Cre^-Sema 3F^F/F^ mice (DLX5/6^Cre^ S3F) compared to other genotypes (**f**, **g**)

We next examined interneuron subpopulations in the hippocampus where a major decrease in NeuN/Nissl-stained neurons within the intervening neurite layers but not the pyramidal layers of the hippocampus was revealed (Fig. 6). Parvalbumin (Parv+), calretinin (CR+), neuropeptide Y (NPY+), and somatostatin (SS+)-positive neurites [56] were quantitated to determine if the cell type ablation is specific for certain interneuron subpopulations. Parv+ neurons and immunoreactive Parv+ fibers decreased within CA1 and CA3 subregions (Fig. 6; black arrows) of DLX5/6^Cre^-Sema 3F^F/F^ but not EMX1^Cre^-Sema 3F^F/F^ mice (Fig. 6a–c). Neuroligin 2 and Parv+/GAD-65 staining of CA3 stratum lucidum is decreased (*p* < 0.001 and *p* < 0.01, respectively) on CA3 pyramidal cells in DLX5/6^Cre^-Sema 3F^F/F^ mice (Fig. 6d, e). Similarly, immunocytochemical analysis of synaptic marker GABA_A_ receptor α1 staining of Parv+ neurons (WT 63 ± 5% vs. Sema 3F KO 43 ± 4%) is decreased (20% [*p* < 0.05], respectively) in DLX5/6^Cre^-Sema 3F^F/F^ mice (Fig. 6). Additionally, Parv+/GAD-65 and Neuroligin 2 stainings of CA3 stratum lucidum are decreased on CA3 pyramidal cells in DLX5/6^Cre^-Sema 3F^F/F^ mice (Fig. 5). In contrast, GABA+ interneurons, GAD-67+ interneurons, and GABAergic/glutamatergic/glial markers were similar in all hippocampal subregions of the EMX1^Cre^-Sema 3F^F/F^ mice (Table 1, Fig. 4, and data not shown).

### Interneuron-Specific Deletion of Semaphorin 3F Produces Social Deficits and Repetitive Behaviors

**Fig. 7:**
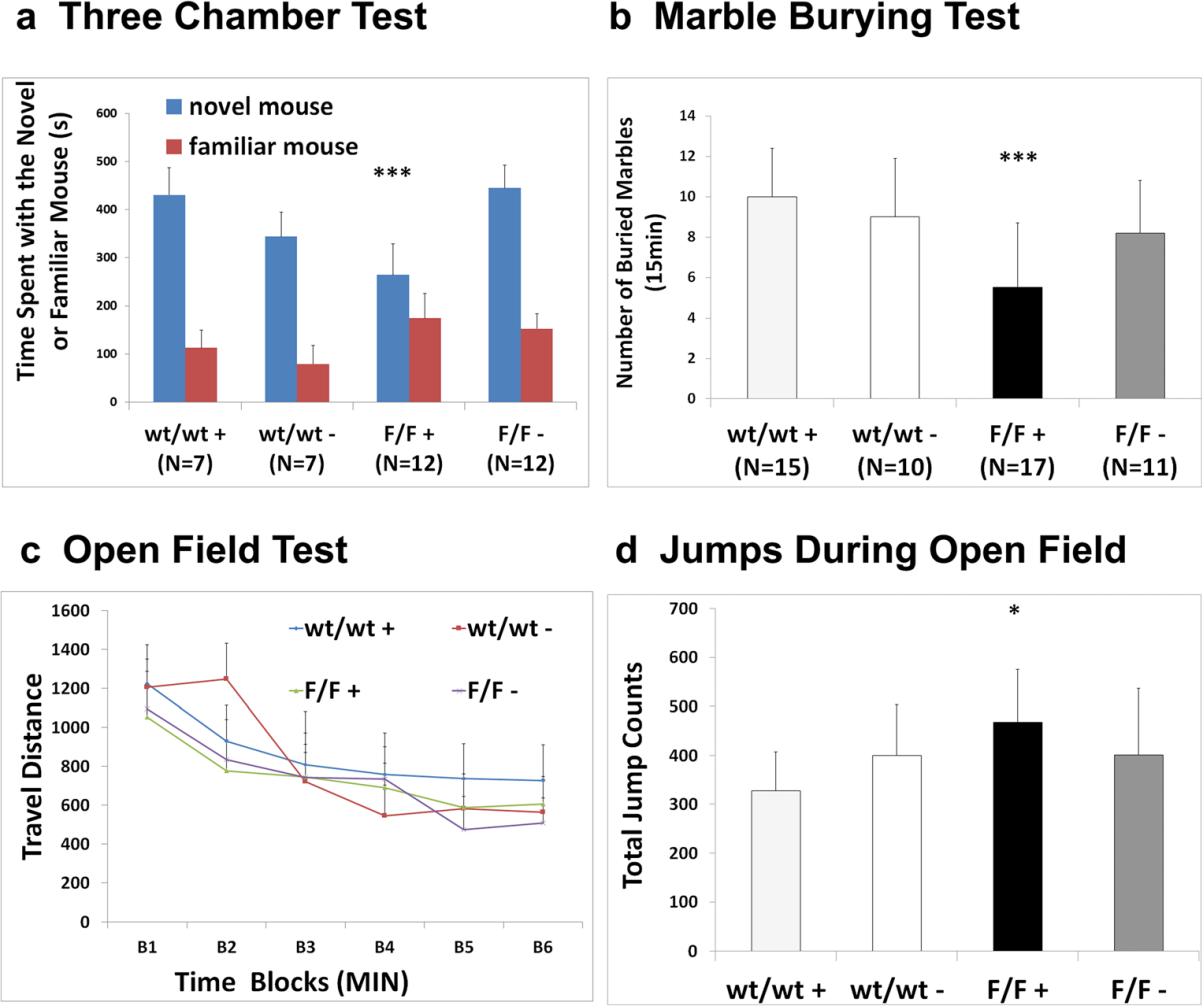
Autistic behaviors in the semaphorin 3F interneuron deletion mutants. Mice in four genotypes, wild type (Wt/WT−), DLX5/6^Cre^ (WT/WT+), Sema 3F^F/F^ (F/F−), and DLX5/6^Cre^-Sema 3F^F/F^ (F/F+), were tested in the three chamber social test (**a**), marble burying test (**b**), and open field (**c**, **d**). The DLX5/6^Cre^-Sema 3F^F/F^ (F/F+) mice had decreased social novelty (*p* = 0.015) and decreased numbers of marbles buried per 15 min session (*p* = 0.0004) compared to other genotypes (**a**, **b**). The DLX5/6^Cre^-Sema 3F^F/F^ (F/F+) mice traveled less time and a smaller distance (*p* = 0.015) compared to the other genotypes (**c**) but had increased jumping (*p* = 0.01) (**d**) in open field testing compared to other genotypes. In home cage testing, we verified the increased repetitive jumping (*p* = 0.01) and repetitive hanging (*p* = 0.05) compared to other genotypes (**d**)

Loss of Sema 3F in interneurons could be associated with autistic-like behaviors. Cohorts of *N* = 8–14 mice per genotype were tested with the three-chamber social interaction test, open field testing, and marble burying test, and the observed naturalistic behavior was videotaped/quantitated in the animal’s own home cage. The DLX5/6^Cre^-Sema 3F^F/F^ mice spent significantly less time with the novel mouse as compared to the DLX5/6^Cre^-Sema 3F^Wt/Wt^ mice in the social novelty test, similar to the constitutive Sema 3F KO mouse [ANOVA, *F*(3, 50) = 7.116, *p* = 0.0004, Fig. 7]. In general, DLX5/6^Cre^-Sema 3F^F/F^ mice spent less time traveling a smaller distance in open field compared to the DLX5/6^Cre^-Sema 3F^Wt/Wt^ mice so activity levels could explain the social novelty test findings [ANOVA, *F*(3, 44) = 3.89, *p* = 0.015]. Repetitive behaviors in the home cage including repetitive jumping [*F*(1, 22) = 7.705, *p* = 0.01] and repetitive hanging behaviors [*F*(1, 25) = 3.933, *p* = 0.05] were increased in DLX5/6^Cre^-Sema 3F^F/F^ mice compared to littermates of the DLX5/6^Cre^-Sema 3F^Wt/Wt^ genotype (data not shown). Similarly, repetitive behaviors in open field testing including trended toward increased repetitive jumping [ANOVA, *F*(3, 45) = 2.21, *p* = 0.09] and jumping were increased [*F*(1, 25) = 6.637, *p* = 0.0163] in DLX5/6^Cre^-Sema 3F^F/F^ mice compared to littermates of the DLX5/6^Cre^-Sema 3F^Wt/Wt^ genotype (Fig. 7). Marble burying behaviors were decreased [ANOVA, *F*(3, 50) = 7.116, *p* = 0.0004] in DLX5/6^Cre^-Sema 3F^F/F^ mice compared to littermates of the DLX5/6^Cre^-Sema 3F^Wt/Wt^ genotype (Fig. 7).

### Deletion of Interneuron Semaphorin 3F Leads to Increased Markers of Oxidative Stress and Inflammation

The intracellular signaling of Sema 3F through the NRP2 receptor regulates the human and rodent MICAL flavoxidases. With the lack of the ligand Sema 3F, we investigated superoxide production, lipid peroxidation, and markers of microglia activation.

#### Hippocampus

**Fig. 8:**
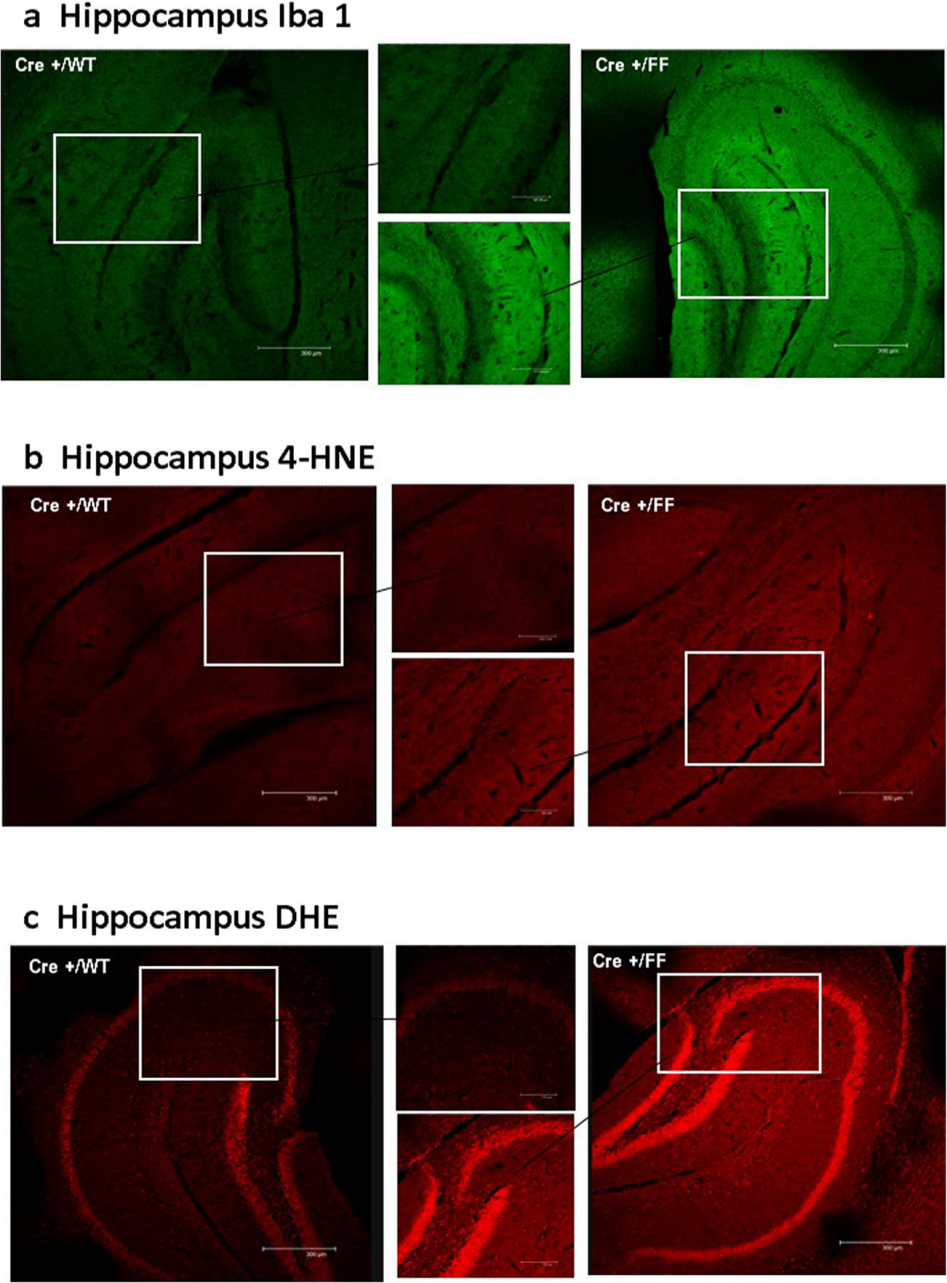
Possible microglial activation and reactive oxygen species production (ROS) in semaphorin 3F interneuron deletion mutants. Mice in four genotypes, wild type (Wt/WT−), DLX5/6^Cre^ (WT/WT+), Sema 3F^F/F^ (F/F−), and DLX5/6^Cre^-Sema 3F^F/F^ (F/F+), were tested via immunocytochemistry for Iba1+, HNE, and DHE. Compared to DLX5/6^Cre^ (+Cre/WT) mice, increased amount of Iba1+ cells, HNE immunoreactivity, and DHE+ cells was observed in the hippocampus of DLX5/6^Cre^-Sema 3F^F/F^ (Cre+/FF) mice (**a**–**c**). Intriguingly, the largest increase in DHE+ cells was in the hippocampal pyramidal cell layers (**c**). Optical density scanning and quantification validated visual observations of increased ROS and possible microglial activation in the cortex, amygdala, and hippocampus of DLX5/6^Cre^-Sema 3F^F/F^ (Cre+/FF) compared to other genotypes and is listed in Table 3. Scale bars in the large pictures are 300 μm and scales bars in the smaller magnification pictures are 125μm

The average Iba1 staining intensity per unit area in the hippocampus of Sema 3F KO mice was significantly higher compared to WT mice (*p ≤ 0.05 Cre+/FF vs. Cre+/WT; Fig. 8a; Table 2). Additionally, there was a clear trend of lower staining intensity when comparing Cre−/FF and Cre−/WT with Cre+/FF that did not reach statistical significance (Table 3). The average 4-HNE and DHE staining intensities per unit area in the hippocampus of Sema 3F KO mice were significantly higher compared to all three controls (***p ≤ 0.0001 Cre+/FF vs. Cre−/WT, Cre−/FF, and Cre+/WT; Fig. 8b, c; Table 3).

**Table 3:** Quantification of Iba1 (microglia), 4-HNE (lipid peroxidation), and DHE (superoxide) staining in the cortex, hippocampus, and amygdala in DLX5/6^Cre^-semaphorin 3F mutants

	Iba1	4-HNE	DHE
Cortex			
Cre+/FF	77.78 ± 29.08	65.36 ± 29.56	81.59 ± 28.11
Cre+/WT	29.08 ± 4.31***	3.25 ± 3.45***	8.79 ± 8.41***
Cre−/FF	30.22 ± 21.90**	7.95 ± 4.77***	8.01 ± 5.53***
Cre−/WT	9.15 ± 9.98***	2.47 ± 5.25***	9.15 ± 4.59***
Hippocampus			
Cre+/FF	38.79 ± 28.28	45.01 ± 17.36	77.08 ± 15.39
Cre+/WT	5.24 ± 6.59	2.85 ± 3.20***	5.02 ± 2.16***
Cre−/FF	18.50 ± 21.09*	3.59 ± 4.47***	5.87 ± 2.11***
Cre−/WT	2.55 ± 2.80	0.08 ± 0.07***	4.45 ± 3.35
Amygdala			
Cre+/FF	44.54 ± 23.01	52.20 ± 25.82	60.33 ± 8.84
Cre+/WT	13.87 ± 18.18*	2.40 ± 3.93***	4.80 ± 3.98***
Cre−/FF	20.73 ± 33.12	1.26 ± 1.85***	4.94 ± 2.20***
Cre−/WT	8.47 ± 12.24*	1.34 ± 1.46***	3.09 ± 1.54***

Optical densities of DLX5/6^Cre^-Sema 3F^F/F^ mice (Cre+/FF) and of controls expressing Sema 3F (Cre+/WT, Cre−/FF, Cre−/WT). Data are expressed as fluorescence intensity per unit area (μm^2^)

*p ≤ 0.01, **p ≤ 0.001, ***p ≤ 0.0001 vs. Cre+/FF

#### Cortex

The average Iba1, 4-HNE, and DHE staining intensities per unit area in the cortex of Sema 3F KO mice were significantly higher compared to all three controls (***p ≤ 0.0001 Cre+/FF vs. Cre−/WT, Cre−/FF, and Cre+/WT; Table 3).

#### Amygdala

The average Iba1, 4-HNE, and DHE staining intensities per unit area in the cortex of Sema 3F KO mice were significantly higher compared to all three controls (***p ≤ 0.0001 Cre+/FF vs. Cre−/WT, Cre−/FF, and Cre+/WT; Table 3).

Finally, there were no statistically significant differences in DHE, 4-HNE, and Iba1 fluorescence intensity between the three control groups expressing Sema 3F in any of the brain regions examined in this study (Cre−/FF vs. Cre−/WT vs. Cre+/WT; Table 2).

## Discussion

Sema 3F–NRP2 signaling has been implicated in pyramidal cell, interneuron, and glia cell function [30, 34, 42]. This study documents the production of a cell-specific interneuron knockout of the Sema 3F gene which results in the reduction of interneuron cell numbers/neurite outgrowth, presumed synapse numbers, and increased excitability plus spontaneous seizures. The interneuron-specific knockout mouse of Sema 3F displays reductions in social behavior and increased repetitive behaviors/restricted interest. Interestingly, this knockout mouse strain has increased immunoreactivity for antigens of oxidative stress, inflammation, and microglia activation. These data suggest the semaphorin–neuropilin interactions in a wide variety of organ systems including the immune system could impact the developing brain and subsequent expression of behavioral phenotypes.

### Semaphorin 3F Signaling Regulates GABAergic Circuitry Formation in the Cortex and Hippocampus

There are two major hypotheses about the regulatory mechanisms that govern the production of interneurons from the ganglionic eminence (GE). Recent analyses of cell fate mapping in the mouse subpallium support one hypothesis that distinct progenitor domains of the GE uniquely generate separate subpopulation of interneurons with each domain correlating with distinct molecular networks [8, 57]. In the other hypothesis, interneuron progenitors within the same regions go through systemic alterations in their potential as development progresses [58]. Regardless of the correct hypothesis, the data from the constitutive Sema 3F and NRP2 mutants plus cell-specific mutants (this report) suggest that Sema 3F–NRP2 signaling functions downstream of genes such as DLX1, Nkx2.1, and COUP-TFII which modulates interneuron progenitor potential [20, 38, 40, 41]. Unlike the Nkx2.1 mutants, deletion of Sema 3F and NRP2 from interneuron does not convert some interneuron subtypes (parvalbumin, neuropeptide Y) into other classes of interneurons (calretinin) [Table 1, Figs. 4 and 6]. Instead, the data are more consistent with the Sema 3F role as a classic guidance molecule in the postmitotic environment, thereby influencing interneuron diversity [42]. DLX1, Nkx2.1, and COUP-TFII can regulate the NRP2 promoter [20, 38, 40, 41]. Sema 3F production by an unknown set of cells in the striatum and cortex prevents invasion of NRP2+ postmitotic GABAergic interneurons into inappropriate layers of the cortical plate or hippocampus [18, 19, 42, 59]. Our data definitely suggest that the interneuron production of Sema 3F is necessary but not sufficient to correctly target some hippocampal and cortical interneuron subpopulations to differentiate appropriately in the correct cell layers.

NRP2 is expressed in precursors located in the ventral ventricular zone as well as tangentially migrating GABA+ interneurons moving to the cortical plate [19, 21, 37]. Since Sema 3F is the ligand for the NRP2 receptor, we used a careful and rigorous immunocytochemical and Nissl stain approach to label and count different excitatory and inhibitory neuronal populations within the hippocampus. While this approach may over- or underestimate actual changes assessed stereologically, the direction and relative magnitude of change across different cell subtypes should be more reliable across the rostral–caudal extent of the cortex and hippocampus. In the DLX5/6^Cre^-Sema 3F^F/F^ mouse, the numbers of GABAergic interneurons, neurite outgrowth, and presumed synapse numbers were significantly reduced in all areas of the hippocampus (Fig. 6) supporting earlier work implicating the Sema 3F–NRP2 signaling system in hippocampal GABAergic migration and differentiation [18, 19, 37, 42]. Regarding migration, the NRP2 receptor ligand (Sema 3F) is expressed during development along the cortical/hippocampal GABAergic neuron migratory pathway, and ectopic expression can alter GABAergic neuron migration [19, 21, 37]. Thus, either cell generation or cell migration during development may at least in part account for the reduced number of GABAergic interneurons seen in Sema 3F-deficient and NRP2-deficient mice (Figs. 4 and 6 [18, 42]). One could not exclude programmed cell death as another possibility. Interestingly, constitutive deletion of the NRP1 receptor did affect GABAergic cell numbers in the murine fetal hippocampus or cortex but adult animals were not surveyed [42].

The main effect of interneuron-derived Sema 3F expression is to influence the proper development of cortical and hippocampal GABAergic circuitry. We found no differences in excitatory neurons within the principal cell body layers (CA and DG) or some glutamatergic markers of the cerebral cortical layers in Sema 3F-deficient mice (Figs. 4 and 5; Table 2). These data suggest in part that the interneuron phenotype is a defect of interneuron-derived Sema 3F expression rather than secondary to effects of pyramidal cells although we cannot rule out this possibility. The data did suggest a 29% reduction in GABA+ cells within the somatosensory cortex of EMX1^Cre^-Sema 3F^F/F^ mice. Similar to defects in NRP2 signaling, parvalbumin (Parv+) interneuron cell numbers, neurite outgrowth, and presumed synapse numbers were significantly reduced in hippocampal subregions (Figs. 4, 5, and 6, Tables 1 and 2) [18]. Thus, our data suggest that conditional deletion of the Sema 3F gene by the DLX5/6^Cre^ transgene affects cell numbers of hippocampal interneurons derived from MGE but less so from the LGE/CGE, as Parv+ interneurons come exclusively from the MGE, whereas most CR+ interneurons come from the LGE/CGE [60]. Alternatively, these cell reductions could be consistent with the cell subtype expression of the DLX5/6^Cre^ transgene. An estimated > 60% of the DLX5/6^Cre^ transgene expression is within either parvalbumin+ or neuropeptide Y+ interneurons [61]. Finally, decreased cortical and hippocampal interneuron numbers could be explained by studies not performed including changes in cell proliferation, birth rates, or cell survival although recent studies would not support this suggestion (Figs. 4 and 6) [42, 59]. Technical limitations of our data preclude making definitive statements about cell migration or generation. However, such limitations do not preclude the importance and novelty of the observation that Sema 3F expression from interneurons at particular GABAergic developmental epochs contributes substantially to the proper development of hippocampal and cortical GABAergic circuitry.

### Semaphorin 3F–Neuropilin 2 Signaling Is a Putative Regulator of the MICAL Pathway and GABA_A_ Synaptic Formation/Function

The deficit Sema 3F signaling in interneurons produces widespread increases in oxidative stress and neuroinflammation across many brain regions (Fig. 8a–c). Multiple hypotheses could account for these data. Sema 3F–NRP2 signaling has a ubiquitous expression in blood vessels and the placenta [62]. Pregnancy factors such as preeclampsia lowers the amounts of Sema 3F expression, but this deficit was not expressed in the placenta of the DLX5/6^Cre^-Sema 3F^F/F^ mouse [12]. The immunologic synapse contains both NRP1 and NRP2 signaling components [63]. Although Sema 3F–NRP2 signaling and its function are poorly understood in the immune system, NRP2 signaling recently is known to affect T cell and endothelial cell function [64]. NRP2 is expressed in microglia and may influence their function [65]. Thus, an immune signaling deficit cannot be confidently excluded at this time to explain the microglial changes and oxidative stress.

A third hypothesis detailing MICAL function downstream of Sema 3F–NRP2 signaling in neurons may better explain the current data in Sema 3F and NRP2 mutants (Fig. 8b, c) [66]. Oxidative stress in parvalbumin interneurons is known to play a role in cellular dysfunction in many animal models of neurodevelopmental disorders including Fragile X, Phelan-McDermid, schizophrenia, and 15q13.3 deletion syndromes [44]. These data suggest that environmental influences during pregnancy may target genetically predisposed interneurons undergoing processes of specification/proliferation, tangential migration, and integration/maturation to facilitate the development of ASD. The Sema 3F and NRP2 gene defects may be a perfect example of how pleotropic signaling may impact the function of one protein (MICAL) across many biological pathways to produce ASD [66]. This deletion produces similar behavioral deficits such as in the NRP2 KO mouse including social behavior (novelty test) and repetitive behaviors (jumping/hanging) (Fig. 7 and data not shown) [28, 29]. Similar to the constitutive Sema 3F KO mouse, the DLX5/6^Cre^-Sema 3F^F/F^ mouse had less distance traveled in the open field, although this may not explain its increased jumping (Fig. 7 and data not shown) [28, 29]. Anxiety was not directly examined in the deletion mouse which could explain some of the observed phenotypes. The decreased marble burying of the interneuron Sema 3F mutant is thought to be related to either decreased attention to novelty or the environment, similar to the restricted interest in ASD (Fig. 7d). These data suggest that the observed behaviors of the DLX5/6^Cre^-Sema 3F^F/F^ mouse and other published Sema 3F–NRP2 mutants result from the functional impact of various neural pathways, including cortical–cortical connections, cortical projection fibers, midbrain dopaminergic projections, and olfactory–limbic connections [67–71]. The cerebellar anatomy was not significantly altered in the constitutive Sema 3F knockout mouse [72]. In summary, the data from the three reported mutants and this report suggest that alterations of Sema 3F–NRP2 signaling produce changes in functional synaptic connectivity, propensity to comorbidities like epilepsy/recurrent seizures (Fig. 3), and autistic-like behaviors [28, 29, 71].

Semaphorin–neuropilin signaling contributes to the development and function of hippocampal and cortical neural circuitry. The signaling localization of the main effector (MICAL) to the cytosol may constrain the defects recognized in these mutants by impacting cytoskeletal function [66]. Cellular processes including cell migration, synaptic pruning, synaptic physiology, neurite guidance and outgrowth, and cell survival are all impacted by MICAL function [73–78]. For instance, semaphorin-regulated MICAL function could regulate the formation, placement, and function of GABAergic synapses (Fig. 6). Semaphorins trigger the rearrangement of the cytoskeleton that usually induces retraction of filopodial or lamellipodia components of growth cones on hippocampal axons and dendrites (Fig. 6) [34, 35]. Deletion of Sema 3F or NRP2 signaling during development leads to increased spine density, decreased dendritic complexity, and abnormal extension of axons (i.e., mossy fibers axons) during hippocampal formation [18, 32–35]. Detailed anatomic, genetic, biochemical, and structural studies (including zinc interactions with NRP2) suggest that Sema 3F–NRP2 signaling is required for cdk5/Fak-mediated dendritic spine formation, synaptic complex elimination, and axonal pruning at excitatory synapses of CA3/CA1 pyramidal cells [34, 35, 79–81]. Electrophysiologic, morphometry, and in vivo epileptogenesis studies in NRP2 KO and here in DLX5/6^Cre^-Sema 3F^F/F^ mice do support a local perturbation in selected synaptic pathways of the hippocampus and cortex (Fig. 3) [18, 33]. Multiple hypotheses could account for these changes, including facilitated excitatory or reduced inhibitory activity. Further, Sema 3A depresses excitatory neurotransmission, while Sema 3F facilitates excitatory neurotransmission on wild-type hippocampal neurons [33]. NRP2 mRNA is mainly expressed in rodent hippocampal pyramidal cells and less so in interneurons [51, 82]. The postsynaptic location of NRP2, as opposed to the presynaptic location of NRP1, and the potential interaction with Sema 3F ligand released from hippocampal interneurons, may play a major role in regulating GABAergic synapse formation, GABAergic neurotransmission, and synaptic plasticity in pyramidal cells, thereby regulating epileptogenesis [33] (Figs. 3 and 6). Our data here squarely extends the knowledge of hippocampal and cortical circuitry formation by identifying hippocampal and cortical GABAergic synaptic development as a putative target of physiologic Sema 3F–NRP2 signaling.

Cell surface expression of GABA_A_ receptors is regulated via a number of mechanisms including axon targeting, trafficking proteins, ligand or second messenger-mediated receptor endocytosis with lysosomal degradation, regulation of protein synthesis, and ubiquitin–proteasome pathway [18, 83, 84]. Emerging evidence suggests that guidance cues such as semaphorins can rapidly regulate the translation of cytoskeletal proteins and possibly other proteins based on whether they are an attractive or repulsive cue through endosomal pathways [85]. Some endosomal pathways require ROS for semaphorin responses [86]. Similar to the report implicating PTEN in Sema 3A-mediated growth cone collapse, Sema 3F signaling in sympathetic neurons and in hippocampal neurons promotes the MICAL-regulated production of superoxides necessary for growth cone function [87, 88] and likely influences both cell migration and cell survival. These MICAL-related processes may regulate the GABAergic cell numbers and neurite outgrowth in NRP2 and Sema 3F mutants (Figs. 4 and 6) [18, 42]. Whether this is a direct or indirect effect of Sema 3F signaling is unclear. Although possible that this is a nonspecific observation of an in vivo experimental system, the specific activation of MICAL pathways both in presynaptic ligand mutants (Sema 3F deficit interneurons) or postsynaptic receptor mutants (NRP2 KO mice) suggests that directed secretion of the Sema 3F ligand from interneurons regulates the MICAL pathway in pyramidal cells and interneurons via NRP2 activation. These data (Fig. 8b, c) suggest a possibility that Sema 3F regulation of superoxide synthesis/degradation rates in pyramidal neurons may indirectly or directly modulate the protein content of GABAergic synapses, thus explaining the observed alterations in GABAergic synapse numbers of NRP2 null and Sema 3F conditional KO mice (Fig. 6) [18]. These changes in GABAergic synapse numbers were not observed in mutants with deficient Sema 3F expression in hippocampal pyramidal cells (Fig. 6). Alternatively, changes in GABAergic synaptic protein content or synapse numbers may reflect a “denervation supersensitivity” adaptation. This adaptive response would cause synaptic excitatory neurons, deprived of their normal inhibitory tone, to alter GABAergic protein expression in an attempt to enhance inhibitory tone and suppress the increase seizure susceptibility [18, 89].

In summary, the Sema 3F–NRP2 signaling system is one of the increasing numbers of pathways where its major targeted physiologic processes are on GABAergic migration, survival, or differentiation [42, 43]. The Sema 3F–NRP2 system also has minor effects on principal cell migration or survival (Figs. 4 and 5) [34]. Sema 3F–NRP2 may have other intracellular targets such as tyrosine receptor kinase or its downstream signaling pathways (mTOR pathway, PI3 kinase signaling) which are located in a variety of cell types including pyramidal cells, interneurons, microglia, T or B cells, astrocytes, endothelial cells, etc. [30, 31, 63, 90–92]. One of these intriguing cells expressing the NRP2 receptor is the microglia which is increased in the Sema 3F interneuron deletion mutant (Fig. 8a) and may play a role in neuroinflammation [64, 93, 94]. Sema 3F–NRP2 signaling has a putative role in T cell migration and blood vessel permeability into the brain [64, 95]. In addition to their role in CNS inflammation, microglia have been implicated in normal neurogenesis and formation of synapses and neural networks during CNS development [96, 97]. Microglia play a role in normal postnatal apoptosis, pruning neuronal connections and controlling the number of synapses and their maturation [98–100]. Microglia may generate ROS as part of these developmental processes. Evidence of oxidative stress and inflammation in the blood/brain of autistic individuals may alter growth of the developing brain, affect serotonin levels, and impair development of neural pathways such as thalamocortical fibers [44–46, 101, 102]. Maternal autoantibodies to downstream components (CRMP1 and CRMP2) to Sema 3F–NRP2 signaling correlate with an ASD diagnosis in children [103]. Further, Wang et al. [104] recently presented evidence that the Sema 3F–NRP2 signaling in cortical pyramidal cells is necessary for postsynaptic homeostatic downscaling. Are pyramidal cells or interneurons the presynaptic source of Sema 3F for this biological process? Deficit GABA signaling in the cerebral vascular endothelial cells (+ for NRP2 signaling) results in defective interneuron migration/differentiation and autistic behaviors in mice [105]. These data impact semaphorin signaling among a variety of cell types including microglia, neuronal subtypes, and endothelial and immune cells. These data suggest future studies looking further at immune–environmental interactions and new approaches to therapeutic intervention in neurodevelopmental disorders. These lines of research demonstrate the critical importance of understanding semaphorin signaling of different cell types during development, interactions with other organ systems, and the potential role that disruption of this pathway plays in autism, epilepsy, and other neurodevelopmental disorders.
